# Hardware Trojans in Chips: A Survey for Detection and Prevention

**DOI:** 10.3390/s20185165

**Published:** 2020-09-10

**Authors:** Chen Dong, Yi Xu, Ximeng Liu, Fan Zhang, Guorong He, Yuzhong Chen

**Affiliations:** 1 Key Laboratory of Spatial Data Mining & Information Sharing, College of Mathematics and Computer Science, Ministry of Education, Fuzhou University, Fuzhou 350116, China; dongchen@fzu.edu.cn (C.D.); n190325013@fzu.edu.cn (Y.X.); x@fzu.edu.cn (X.L.); n170320073@fzu.edu.cn (F.Z.); n170325003@fzu.edu.cn (G.H.); 2 Fujian Provincial Key Laboratory of Network Computing and Intelligent Information Processing, Fuzhou 350116, China; 3 Key Lab of Information Security of Network Systems (Fujian Provincial), Fuzhou 350116, Fujian Province, China

**Keywords:** hardware security, integrated circuit, biochip, neuromorphic computing, Hardware Trojan, protection technique, detection technique, prevention technique

## Abstract

Diverse and wide-range applications of integrated circuits (ICs) and the development of Cyber Physical System (CPS), more and more third-party manufacturers are involved in the manufacturing of ICs. Unfortunately, like software, hardware can also be subjected to malicious attacks. Untrusted outsourced manufacturing tools and intellectual property (IP) cores may bring enormous risks from highly integrated. Attributed to this manufacturing model, the malicious circuits (known as Hardware Trojans, HTs) can be implanted during the most designing and manufacturing stages of the ICs, causing a change of functionality, leakage of information, even a denial of services (DoS), and so on. In this paper, a survey of HTs is presented, which shows the threatens of chips, and the state-of-the-art preventing and detecting techniques. Starting from the introduction of HT structures, the recent researches in the academic community about HTs is compiled and comprehensive classification of HTs is proposed. The state-of-the-art HT protection techniques with their advantages and disadvantages are further analyzed. Finally, the development trends in hardware security are highlighted.

## 1. Introduction

With the rapid development of Cyber Physical System, the popularity of electronic devices are getting higher and higher. In daily life, people use electronic devices to shop, transfer money, take videos, and record information. In company and bank, the data are processed and stored by electronic equipment. Even in military defense, electronic devices are also inevitably employed. The smart earth, smart city, industry 4.0, intelligent robots, autonomous vehicles, etc., which people will realize in the future are all products of highly used electronic devices. It can be said that how convenient is people’s lives and how much people rely on electronic devices.

Unfortunately, like software, the hardware still has security risks, while people had not been aware of hardware security issues for a long time. In recent years, experts and scholars have carried out a certain degree of research on hardware security, especially the security of chips, which are the core part of the hardware. As we know, the whole design and manufacture process of the chip is very complicated. To get the most profit, several third-parties are involved, such as Intellectual Property core (IP core) suppliers, Electronic Design Automation (EDA) software suppliers, and manufacturing factories. Under the guidance of Moore’s Law and Dennard Scaling, the integration of ICs is booming. Hundreds of millions of gates are accommodated in a single chip, called the Very Large Scale Integration (VLSI) circuits, the Ultra Large Scale Integration (ULSI), the System on Chip (SoC), even the Network on Chip (NoC).

Obviously, this offshore foundries technology brings high-security risks to the entire electronics manufacturing industry. As the scale of ICs grows rapidly and manufacture mode becomes more flexible, the main security issues of ICs are caused by the implant of the malicious circuits, named Hardware Trojans (HTs) [1]. The development trend of the ICs results in them extremely vulnerable to attack by HTs [2].

The standard definition of the HT was proposed by the IBM Research Center in 2007 [3]: the HT refers to the malicious circuits or harmful alterations to the original circuit that exists from the chip design stage to the chip packaging test stage. The HT lurks in the dark and makes changes in the design stage of the IC [4].

**Figure 1:**
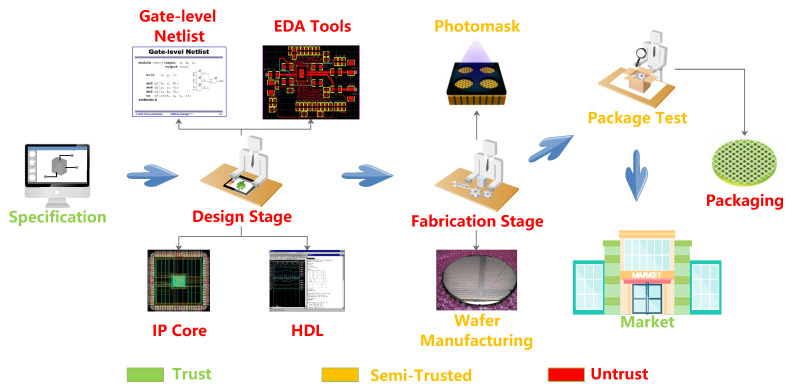
The credibility level of a modern IC life cycle.

Figure 1 shows the credibility level of the IC, depending on various stages [5]. The production line of ICs is mainly divided into three parts: design, fabrication, and testing [6]. When compared with the IC fabrication stage, there are many ways to implant HT in the IC design stage. These would cause some undesigned problems, such as modification of the function, DoS, and the leakage of critical information [7].

The green parts of the Figure 1 are the trusted stages, including three parts: specification and market entry. In these stages, the attackers cannot insert the HTs. The red parts occupy half of the process in Figure 1. At these stages, HTs are extremely easy to be inserted due to the pipeline operation. The semi-trusted stages of the orange part will bring the attackers some troubles, but some HTs can be inserted, so it is called semi-trustworthy. Last year, Xue et al. suggested that there was also the possibility of falsifying the test results and concealing the existence of HT in the testing stage [8].

As early as 1991, the US military activated “Trojans” that were installed in printer chips, the enemy’s air force is constrained [9]. Syria had suffered an air strike more than a decade ago, due to the radar chips implanted in the HT [10]. In 2017, Garg pointed out that the outsourcing IC components have a risk of trust, his paper also expressed concerns about the security of the finished IC [11]. It is conceivable that the security issues of the IC will also grow dramatically. In short, the prevention of IC security is urgent [12].

In the future, the influence of HTs may extend to unknown areas, and even trusted fields in the past. The challenges of dealing with HTs mainly include the following four aspects: (1) It is difficult to adequately distinguish the influence of process noise disturbance with HT because of the small scale. (2) The activating condition always is a rare event, it is impossible to predict when the HT will be executed. (3) Owing to numerous types of HT, it is difficult to defend them with a unified method. For example, because of structural differences, digital circuits and analog circuits can hardly detect the HT of both in the same way. (4) With the diversification of IC design concepts [13,14,15,16,17,18,19,20,21] and the continuous expansion of scale, the HT of corresponding chip platform will become more complicated and its attack patterns will also be diversified.

Obviously, from the above four aspects, the prevention and control of HTs is a tough problem, and it will be a persistent tricky problem in the future. It is extremely urgent to study how to detect the hidden HTs in ICs quickly and precisely, ensuring hardware security.

The existing HT researches have yet to be increased and there is still considerable potential in this research field. In practical applications, the impact of HTs is receiving attention [7]. Some articles designed or considered some potential HTs from the attacker’s point of view and put forward solutions to these HTs [22,23,24,25,26,27,28,29,30,31,32]. Some researchers applied HTs to the real environment and designed a scheme to defend it [33,34,35,36,37,38,39,40,41,42,43,44]. Individual scholars proposed methods to reduce the effect of the HTs [44,45].

However, a deep and macroscopic understanding is more important for dealing with HTs. Only based on a comprehensive understanding, catching the HTs’ characteristics to defense them. The previous literature has some HTs reviews, which has some limitations when compared with this article. Huang et al. [46] discussed in detail many HT detection and prevention techniques that are based on machine learning (ML). However, the credibility model is a bit outdated, and the testing stage is not revised to a semi-trusted state. Additionally, the detection objects discussed are more limited to digital circuits, ignoring the possibility of applying ML on biochips. Moreover, the detailed discussion of HTs safety on RF IC and AMS circuits is not included in this article. Sumathi et al. [1] studied the attack and defense of HTs on programmable logic devices (PLD) and application specific ICs (ASIC). Nonetheless, the characteristics of HTs have not been classified. What is more, the HT detection methods were emphasized, and the prevention technologies have not been discussed in a framework. Alam et al. [47] included the HTs for the analog circuit in the review, and compared to the digital circuit HT, it further expanded the discussion of HT in the IC category. If this paper can increase the HT research discussion about emerging chips, the review will be more complete. Sidhu et al. [48] completed framework work on the classification, detection, and defense for HTs. Otherwise, there is a lack of cutting-edge detection methods (such as ML-based detection methods), and there is no precise division from 2D to 3D in the split manufacturing for design. Besides, the various detection and prevention technologies have not carried out detailed comparisons about their advantages and disadvantages, and the scope of discussion for HTs has not been expanded to other non-IC chip platforms. Did not carry out a complete open issue work. Because of the shortcomings of the above papers, this paper makes the following contributions:

- Considering the attack features and destructiveness for HT, the threats are summarized into the framework and divided into four layers: Device layer threat, System layer threat, Data layer threat, and Application layer threat. The threat level is progressive.
- Proposing some descriptions of HT’s specific attack on the three platforms (Biochip, AI chip, and AMS/RF IC). Due to the particularity of these chip platforms, the physical structures for HTs are different accordingly. For instance, HTs hide in the memristor, new-style components, and in AI chips. Completing HT classification based on different chips.
- Summarizing the future research directions of HT. Especially predicting golden-free and ML detection technology that will be the future study directions, for the zero-overhead tendency of future HT design. At the same time, this paper emphasizes the benefited application for HTs.

The rest of the paper is organized, as follows. In Section 2, according to the threat level of HT, the corresponding layers threat framework is proposed. In Section 3, expanding the description of attack characteristics of HT on some emerging chip platforms. HT classification description for different properties in Section 4. Compiling the recent literature about HT, proposing the classification of trojan detection/prevention technologies in Section 5. Section 6 thinks about the change direction of future HT design and the tendency of its detection technology. Section 7 presents the conclusion.

## 2. Hardware Trojans Threat Framework

**Figure 2:**
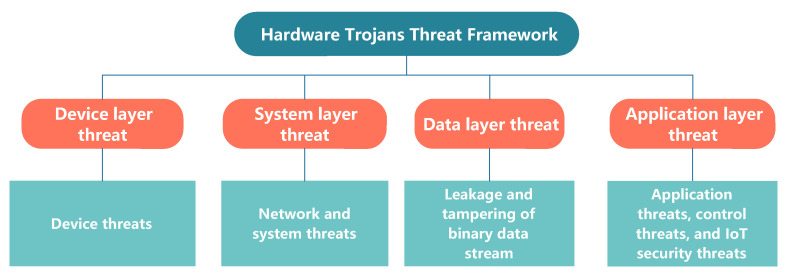
Definition of the HT threat framework in the respective four layers.

In this Section, the paper refers to the information security level framework constructed by Yin et al. [49] and combines the objects, characteristics, and cases for HTs threats to propose the HTs threat framework. The framework is divided into four parts: device layer threat, system layer threat, data layer threat, and application layer threat. Below, the paper directly divides them into four subsections to explain them in turn. Figure 2 shows the threat definition at each layer.

### 2.1. Device Layer Threat of Hardware Trojans

Device layer threat is defined as device damage that is caused by HTs. Similar to the fact that information depends on a physical carrier, HTs are presented on the chip in the form of additional physical implants or tampering with hardware parameters, and the chip relies on the device as a carrier. Therefore, malicious tampering with chip hardware means that HTs cause physical damage through the device. This physical destructiveness is often invisible on visual inspection but illustrates the concealment of HT. This kind of implantability or parasitics best reflects the threat features at this layer, since the bottom layer of the circuit device is the primary goal of HT implantation. The reliability HT could even directly damage the components of the IC device itself, based on the two principles of negative bias temperature instability (NBTI) and hot carrier injection (HCI) to accelerate transistor aging [50]. When compared with implantation and parasitic threats, the time-sensitive threat that is based on reliability HT is even more harmful. Device layer threat is the most widespread type of HT threat, which also leaves hidden dangers for HT information attacks on the device layer. Threats at the device layer reflect the security requirements of the hardware carrier.

### 2.2. System Layer Threat of Hardware Trojans

The system layer threat manifests as interference to network and system. Starting from this layer, the destructiveness brought by HTs has been enhanced. Software trojans have similarities with HT, which is, they are both camouflaged. Software trojans are malicious codes that are parasitic on software programs, but, on the surface, they are consistent with normal software. The user enters private information, such as account number and password, into the infected malware window, and the attacker remotely controls the software to illegally receive the data. When certain trojan programs respond to user requests, they continue to pop up error prompt windows to DoS, resulting in failure to complete normal program use. There have not been many examples to show the damage to the software system due to the insufficiency of detection technology at this stage and the limitations of HT design methods. However, with the development of technology and the deepening of research in the future, the malicious influence of HTs on software systems is not impossible. Boraten et al. designed a target-activated sequential payload (TASP) HT on NoC, which, in conjunction with a counter based on the finite state machine (FSM), causes irreversible failures on multiple links. The adversary could further rely on these errors to send numerous data packets, causing link congestion and even the entire network failure [51]. The superposition of multiple HT attacks can be an opportunity to cause damage to the hardware system, and it has become possible to cause harm to the network with the help of HT with specific components.

### 2.3. Data Layer Threat of Hardware Trojans

The data layer threat can generally be understood as the risk of attacks on data, identity, and privacy information. From the root cause, it is reflected as HTs leaking and tampering with the integrity of the binary stream data on the original circuit. A simple HT that is based on electromagnetic leakage was a case of data layer threat [52]. When HT was activated, the acoustic signals were used to express the bits of the key, and then radiated out by radio wave. An attacker used an ordinary radio to receive these signals, interpreting the key through the different sounds expressed by these signals. The adversary implants HT backdoors to attack the biochip used in medical diagnosis, and the identity information and privacy information of the patient’s condition can be easily obtained illegally through reagent residues. There is an example of HT on the new-style artificial intelligence (AI) chips [53]. Due to the infection of HT, the ML classifier on the chip has been slightly tampered with the parameters of the image samples, producing adversarial samples [54] that are invisible to the human eye, making the ML classifier produce erroneous results. Even HTs rarely output abnormal signals, which may sometimes be confused with hardware malfunctions.

### 2.4. Application Layer Threat of Hardware Trojans

Application layer threat emphasizes application threats, control threats, and IoT security threats. A more obvious feature of threats at the layer is the security threats generated under the influence of interaction with end-users. Therefore, in the IoT, smart devices, driverless cars, and robots that rely on SoC devices [55] cannot do without sensors to sense operations. If they suffer from HT security vulnerabilities, they will put other devices in the shared network at the same risk. Even chips with similar loopholes on atmospheric observation radars [56,57] produce the same destruction. Hackers only needed to launch an HT attack on the joint test action group (JTAG) interface on the printed circuit boards (PCB) and illegally accessed the memory through the data bus to obtain the authorization to modify the data [58]. On NoC, there was a kind of HT that can launch bandwidth DoS attacks. This attack phenomenon slowed down communication, decreased application performance, and affected system reliability [59]. The above three cases illustrate the concept of application threats for observation interaction, interface interaction, and communication interaction. However, application threats are not limited to these three approaches. Any interaction approach that can realize the threat can be incorporated into this concept. An analog HT, called A2, could remotely steal software control rights to achieve the attack purpose, which indicated the control threat [60]. Generally, the HT’s aggressiveness at this layer cannot be underestimated.

## 3. Hardware Trojans on Special Chips

In recent years, the chip industries have developed extremely rapidly and the style of chips is no longer restricted to traditional circuits. New chips, such as analog/mixed-signal (AMS) and radio-frequency integrated circuit (RF IC), Biochip, and AI chip have appeared one after another, in order to meet the current targeted and diverse needs. However, the impact of HTs is slowly spreading to these emerging chips.

### 3.1. AMS/RF IC

The structure of the analog/mixed-signal (AMS) circuit is different from the digital circuit. It has analog and digital components, the radio-frequency integrated circuit (RF IC) belongs to the category of the AMS circuit. The combination of the trigger and the payload in the AMS circuit can be more diversified, which can be an analog trigger plus a digital payload or an analog trigger and an analog payload [47]. Oscillators, filters, bias generators, and opamps are the targets of HTs.

Transistors are the components that make up the AMS. Unlike digital circuits that have numerous logic gates, the AMS is only less than a hundred or so. Once the HT is implanted on the AMS circuit, the layout will be reorganized, so it is not necessary to discuss the HT in the fabrication stage. The analog circuits in wireless networks are easily accessible to HTs. Since HTs do not require physical access to the circuit, they just rely on a public wireless channel to communicate. The more serious problem is that the process variation caused by the mismatch of the analog circuit device [61], which provides living space to HTs. However, the process variation is a natural phenomenon during the chip fabrication stage [62]. For adapting to the changes in the manufacturing process or operating conditions, some parameters of the analog characteristics for the wireless transmission (such as amplitude, frequency, phase, etc.) are defined as a variable value [63]. Unfortunately, the enormous process variation can not only lead to the injection of HTs, but also makes it impossible to detect HTs due to the measuring noise and the environmental changes. Untrusted IP and malicious designs should also be considered in the attack model. There are not many HT detection methods for AMS circuits at present, even there is not effective detection technology to overcome HT with analog trigger. The detection technology for such circuits in the future needs further development.

**Figure 3:**
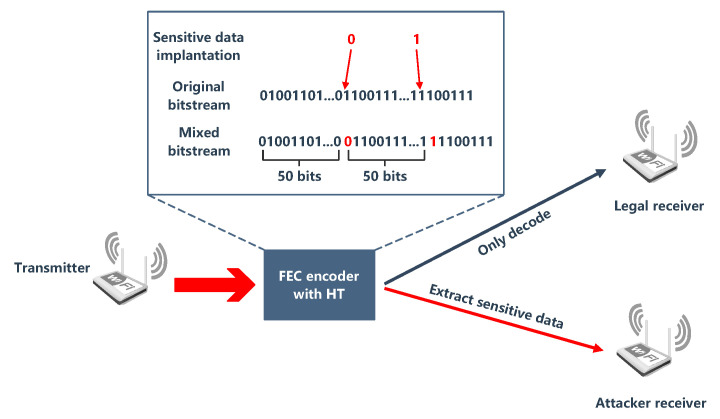
Threat model of forward error correction (FEC)-based Hardware Trojan (HT) in the wireless network.

RF ICs are widely used in many wireless network environments, and some algorithms [64,65] to optimize throughput have promoted the performance of wireless devices, which also provides opportunities for HTs. For example, the forward error correction (FEC) encoder in the wireless routing of the transmitter is implanted with HT. The correct bit will be maliciously replaced during the transmission of information, so that the receiver may receive incorrect information. Figure 3 shows the principle of FEC-based HT attack. Although such malicious changes will cause slight changes to the signal-to-noise ratio (SNR), the receiver only treats them as a normal noise interference [66]. In another case, there are usually multiple wireless interfaces (WIs) on the wireless network chip. The HT on the interface will interfere with the signal for a long enough time, making the calibration/detection module ineffective. This situation will evolve into a DoS attack and even other WIs on the chip will be affected [67].

### 3.2. Biochip

Biochip is a microfluidic device, called a lab-on-a-chip (LOC). Today, when conducting biological experiments, it is difficult for experimenters to manually extract, classify, and mix trace amounts of chemical reagents. Biologists use reagents to complete a series of biochemical reactions on biochips, which can efficiently obtain new reagents and avoid waste. The chip operates with a code program (Biocoder) and computer aided design (CAD) in a computer; biocoder is regarded as a biological protocol. The design of biochips and the construction of components are extended from ICs. The HT safety on biochips can be discussed, according to the same idea of this analogy. Despite the increasing research on the design of biochips, researchers knew very little about the specific implementation methods for HT attacks on biochips, and more often mentioned possible attack scenarios [68].

**Figure 4:**
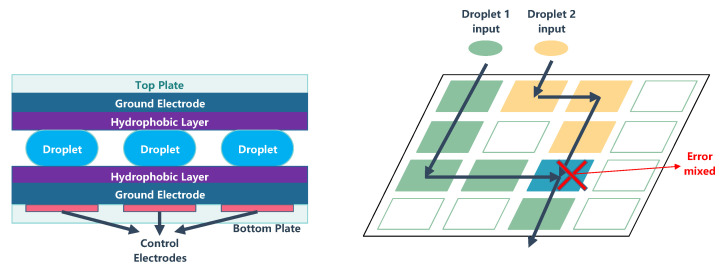
Schematic diagram of the digital microfluidic biochips (DMFB) structure and path error.

Like ICs, biochip suffer malicious risks at the fabrication stage [69]. In the process of chip production, they implant HTs to manufacture malicious biochips, so that the flow path of the droplets, as shown in Figure 4. Wrong or unexpected droplet mixing tend to the destruction of biological experiments and waste of reagents [70]. Some biochips based on the BioMark HD platform (a biochip technology) monitor the flow of droplets with a charge-coupled device (CCD) camera [71], the microcontroller is the core device.

**Figure 5:**
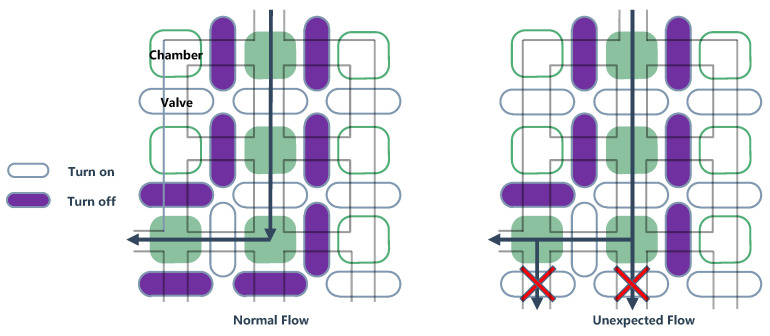
Normal flow and unexpected flow in the field-programmable gate array (FPVA) biochip.

A famous biochip expert pointed out that, if the above two devices are injected with HTs, the chip’s security monitoring and normal operation will make a lot of trouble. Even the micro-electrode-dot-array (MEDA) biochip [72] leaves room for HT implantation in hardware layer. The valves in the flowbased microfluidic biochips (FBMB) [73,74] and the field-programmable gate array (FPVA) are key devices. When the hardware is threatened, it may accelerate the valve aging or cause incorrect switching and contaminated reagents (Figure 5 demonstrates the process of reagent contamination, the gray line represents the liquid pipeline). In a word, the research for the HT in biochips is worth further exploration.

### 3.3. AI Chip

Neural network algorithms has driven the advancement for image recognition and detection [75,76,77,78,79,80,81]. Meanwhile, artificial intelligence (AI) chips that are based on these ML algorithms have brought a further breakthrough in the computing performance for traditional ICs. AI chips can be divided into two categories, traditional AI chips and new-style AI chips [82].

Traditional AI chips still use CMOS components to carry out the calculation of neural networks. Among them, field programmable gate array (FPGA) and ASIC platforms are representative of such AI chips. Threat models of HTs that previously existed on FPGAs and ASICs also appeared on such AI chips, since they have the same hardware environmental. Not only it is vulnerable to the HT in third-party intellectual property (3PIP), but it is also easily implanted into HT by malicious designers at the design stage. There are too many examples, and the subsection will not repeat them here. The new-style AI chip uses a new device (memristor) such as non-volatile memory. They can greatly reduce circuit overhead and improve computing efficiency [83]. However, the emerging chip is beginning to be at risk from HT attacks.

**Figure 6:**
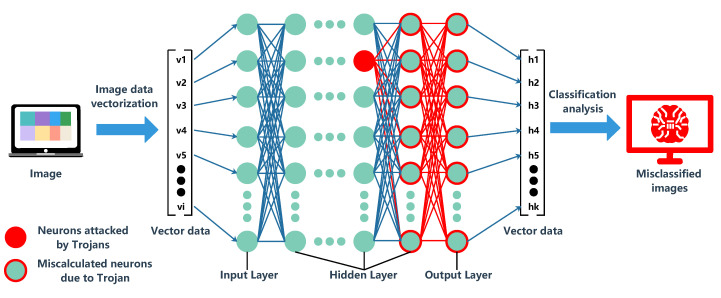
Threat model of neurons attacked by HTs.

The attacker injected a small HT circuit into the neural network, which made a change for the output parameters in the hidden layer. At the same time, it led to subsequent calculation errors. Figure 6 shows that the neuron attacked by HT in the AI chip modified the classification result of ML [53]. Even the HT hides in the memory controller that interacts with the neural network accelerator. An input image may activate the trojan and the final classification accuracy will be affected [84]. If a trigger is implanted in the memristor, then the pulse signal in the circuit may activate HT under the influence of signal delay or voltage change. DoS and fault injection are inevitable [85]. The security risks on these AI chips deserve further attention.

## 4. Hardware Trojans and Their Classification

The paper processed this part about the basic structure of HTs and their classification. Many previous papers have given the most basic structure, they do not cover all of the mainstream HTs at present. Based on the structure of the HTs and recent papers, the section added some content to the most general classification so far.

### 4.1. Structure of Hardware Trojans

The fabrication stage is in a untrust position, but HTs are hardly inserted at the photomask and the wafer manufacturing stage. In IC design stage, the designers treat chip components as modules for layout, and then complete wiring arrangement for each module. In the process of module layout, the attacker may insert extra space into the chip as a module of HT. The HT lead to changes in current and power consumption.

**Figure 7:**
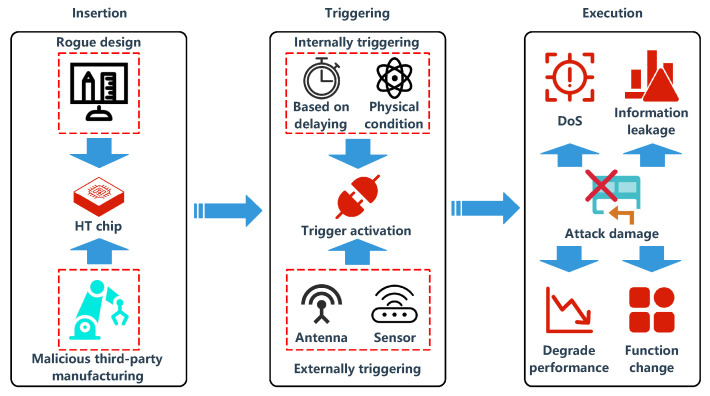
Insertion, triggering, and execution for HTs.

**Figure 8:**
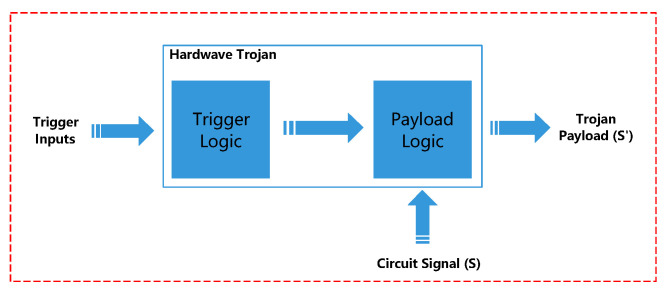
General structure of a HT in a design.

**Figure 9:**
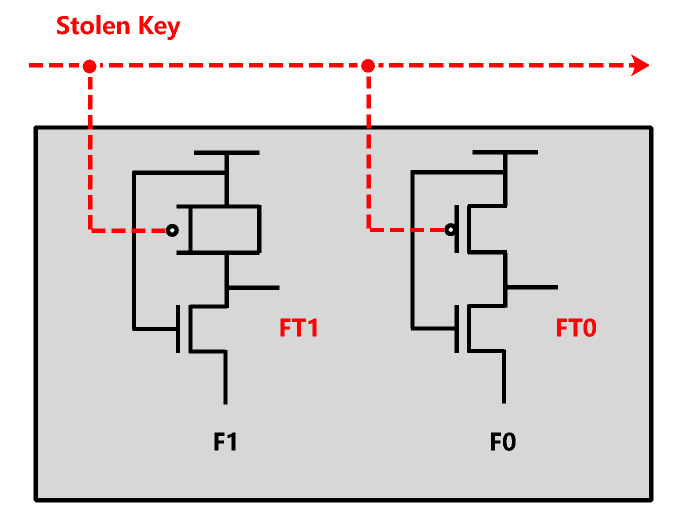
A special HT without trigger.

Furthermore, in serious cases, it will make the logic function fault. The delay time of the signal, chip area, and chip weight will be inevitably changed. The variety of HTs is numerous, they have an unfixed structure, various attack means, and effects. Some HTs will leak important confidential information through the built-in backdoors. Some HTs did not have any impact on the circuit itself, but provide a back door for the malicious software to attack the system. Some HTs will seriously affect the normal operation of the system, destroy the hardware facilities, and bring security threats to the information assets of the country [7]. In general, HTs have a complete process from implantation to trigger execution. Figure 7 contains the attacker objects, trigger conditions, and execution destruction effects in the process. Figure 8 is the operation principle diagram of a typical HT, which shows the circuit signal change process [86]. Usually, the composition of a common HT is elementary, consisting of a trigger block and a payload module, but not all HTs have the triggers, such as the HTs in the AMS IC [36], as shown in the Figure 9.

This simple structure of the HT only contains two PMOS transistors whose flip-flops depend on the primary circuit. The trigger module receives a unique circuit parameter signal. After processing, the payload module outputs the result. For a simple HT, it is highly concealed and destructive. These HTs keep silent most of the time due to their rare activating conditions. Therefore, it is difficult to be found. The specific signals that activate HT do not normally appear unless the circuit is running long enough. The effects of maliciousness are inevitable [87] once HT is triggered.

### 4.2. Classification of Hardware Trojans

**Figure 10:**
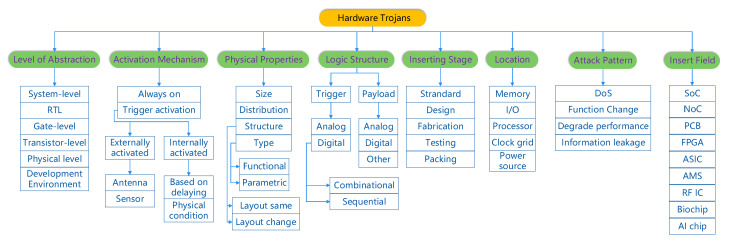
Comprehensive classifications of HTs.

The researchers [88,89,90] divided the HTs from its basic characteristics into three categories: Physical characteristics, Activation characteristics, and Action characteristics. The above three points correspond to the physical properties, activation mechanism, and attack pattern of Figure 9, respectively. Additionally, they proceeded extension classification based on it. Refs. [91,92,93,94] classified the HTs according to the structure of HTs into two major categories: Trigger, Payload. It could be subdivided into digital type and analog type. Based on the previous classification, Refs. [7,95,96] gave a comprehensive taxonomy, including the logic type, functionality, and so on. There is a relatively complete taxonomy of HTs. With the rapid development of HTs, its classification standards are also rapidly changing. Hence, the paper synthesizes the experience of predecessors, giving the newest classification of HTs, affording reference for other researchers. Figure 10 shows the detailed classification.

#### 4.2.1. Inserting Stage

The fabrication process is not safe due to third-party involvement, including various manufacturing tools and outsourced manufacturing. HTs can insert each level of the process and interfere with the operation [90,97]. If the chips are completely protected from HTs, a manufacturer alone undertakes the entire chip production line, but the cost is not economical.

#### 4.2.2. Level of Abstraction

It contains six levels: System-level, RTL, Gate-level, Transistor-level, Physical-level, and Development environment. System-level is the highest level of abstraction. In this level, all of the text descriptions required by the designers will be finished, but an HT may be inserted by changing the protocols among modules, owing to the lowest complexity. The Register Transfer Level (RTL) is relatively dangerous, and most of the signal functions will be stored in the register. Once the attacker has mastered the hardware, it can greatly increase the insertion probability of HT, and no level can be spared [97]. In the Transistor-level, attackers can insert an HT by modifying or replacing the transistor.

#### 4.2.3. Activation Mechanism

Using devices, such as antennas or sensors to remotely control the HT from the external environment, the method belongs to the externally activated attack. The always-on HTs are relatively rare because they have hardly concealment. For the aggressor, hiding the HTs is the most significant. Generally, the HTs keep inactive. When coming to some conditions, such as receiving a unique signal or operating in specific states, the HTs are activated. It is difficult to estimate the impact of HT before it is triggered, which proves that the malicious consequences of HT are difficult to measure [98]. This is the idea behind the majority of today’s HTs.

#### 4.2.4. Location

A complete circuit contains several components, such as CPU, Memory. The HTs in the Processor can change instruction or execution cycle; The HTs in the memory can modify the address or stop some operations to the memory; The HTs in I/O can leak the input or the output to the attackers, even modifying the original data of an operator. The HTs in the power supply can affect electric signals, making the device abnormal. Finally, HTs cause the clock frequency to change and the clock signal will not be released properly [7].

#### 4.2.5. Attack Pattern

The HTs can be classified according to their effects, such as information leakage, DoS, etc. [95]. Information leakage is destructive to military actions and commercial activities, which even can destroy a country or an enterprise. Information leaking in HTs exists in neglected wireless network equipment, such as RF, AMS, etc. In the HTs in I/O mentioned above, the attack of DoS can use up all sources in the circuit and disenable the device to work normally. The HTs tampered functionality can impact the function, causing subtle differences in the output.

#### 4.2.6. Physical Properties

Based on the physical characteristic, the HTs can be divided into four categories: Size, Structure, Type, and Distribution. In terms of the Type, the HT includes the functional type and parametric type: functional HTs run its function by adding or removing transistors/gates; parametric HTs run its function by modifying wire thickness or other possible circuit parameters [96]. On the one hand, some HTs may have different size with the same purpose. On the other hand, some HTs have the same size with various functions, which made testing HTs harder.

#### 4.2.7. Logic Structure

In the previous introduction, the simplest HT consists of the trigger and payload [93]. The trigger type can be divided into digital type and analog type. The former is usually activated by the boolean logic function, but the latter is caused by various analog conditions. Based on the logic type, the digital type can again be classified into the combination type and sequential type. The former is activated when the internal signals of the circuit come to a certain value. The latter needs a continuing exchange of states. For instance, in FSM, the attacker mixes malicious signals and normal signals, the effect that is produced by HT is more covert. The payload type can also be mainly divided into the digital type and analog type. Apart from the above two types, other types of payloads are few and rare. The subsection does not discuss them as the mainstream.

In this paper, the subsection combines some past classification ways. The reason for adding the subsection is that it is also a vital category that appeared in many previous articles. The paper considers that Figure 9 can stand for the 99% of the HTs in the market, and the other 1% is for where there may be some very special HTs the paper never discover. Actually, it is relative with some items in the classification figure.

#### 4.2.8. Insert Field

No matter the traditional SoC and NoC, or the advanced RF IC and AI chip, you can find the trace of the HTs. On SoCs, HTs can bring untrusted threats to 3PIP, because the influence of HT, NoC will be attacked by information leakage and DoS. The traditional AI chip mainly comes from FPGA and ASIC [82], and they are also fact with the risk of IP theft. The more advanced AI chips have a key component-memristor, which is easy to become the target of HTs. The HT on the RF IC can steal one bit in turn to complete the key leakage during the key transmission process [36]. The HTs in AMS circuits have a very strong concealment. Usually, they do not occupy any overhead of the circuit and are hidden in the positive feedback loop of the circuit. In this way, they can constantly interfere with the amplitude and frequency in the circuit to produce result with considerable damage. HTs can even change the signals of components, such as resistors in PCB circuits.

## 5. Hardware Trojans Protection

The paper considers both detection and prevention as a protection of the ICs. The detection techniques are developing in a vague direction. Diversified detection and prevention technologies ensure the possibility of coping with multiple types and multi-platform HT. This section mentions these technologies.

### 5.1. Classification of HTs Detection Techniques

HTs with high concealment, small area overhead, and simple design are becoming increasingly common. Additionally, the design concept of HTs is developing towards zero overhead, intelligence, and decentralization. Meanwhile, the HT detection techniques are also constantly advancing, but they are still in primary stage. The current HT detection techniques refer to determine whether HTs are included in the circuit.

So far, some researchers have put forward countermeasures for HTs [26,87,91,93,99,100,101]. Xiao et al. [99] divided the measures for HTs into three categories: HT Detection, Design for Trust, and Split Manufacturing for Trust. At present, the classification of this article is relatively complete. The HT detection category can be sub-divided into Post-silicon (before the finished chip) and Pre-silicon (after the finished chip). The Design for Trust category can be subdivided into Facilitate, Prevention of HT Implantation, and Trusted Computing. The Split Manufacturing for Trust can be subdivided into 2D/2.5D/3D Manufacturing [99]. Similar to that article, Bhunia et al. [93] classified the detection techniques into HT Detection Approaches, Design for security, Run-time Monitoring. Dupuis et al. [87] referenced the [99] and scaled back it. Refs. [91,100] divided the hardware detection techniques into nondestructive and destructive. These papers were subdivided into invasive and non-invasive. The invasive category stands that the testers modify the design of ICs and implant some devices into the ICs to assist in detecting the HTs. The non-invasive category stands that the original design of the ICs is never altered. Rostami et al. [101] did not make a classification for detection technology, but they enumerated some possible attack targets and developed measures to address these problems. Li et al. [26] utilized the market model to express the status and threat of HT.

**Figure 11:**
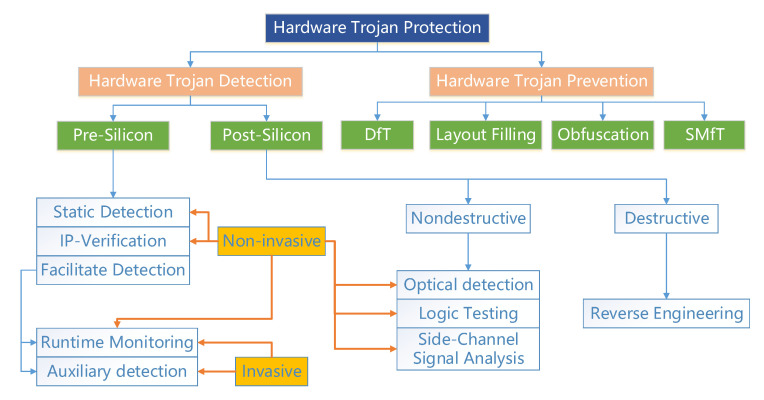
HTs detection techniques classifications.

The current mainstream of the detection techniques has been comprehensively classified, as shown in Figure 11. According to the production process of ICs, the mainstream IC detection is classified into pre-silicon and post-silicon. The following subsection discussed each detection technology based on these two branches. Figure 11 demonstrates the destruction properties and invasion properties for these technologies.

### 5.2. HTs Detection Techniques in Pre-Silicon

#### 5.2.1. Facilitate Detection

Facilitate detection mainly deals with the rare activating nets, process variations, and measurement noise, which benefits some detection technologies. It can be divided into Auxiliary detection and Runtime Monitoring.

- **Auxiliary detection**

Auxiliary detection is a means of further enhancing the effectiveness of logic testing and channel analysis. Through properties’ comparison between HT-free IC and infected IC, auxiliary detection serves side-channel signal analysis by comparison of the result amplification. The application of logic testing should be based on the fact that the circuit is difficult to be invaded by the HT, and the condition is difficult for the opponent to grasp [87].

Salmani et al. [102] developed a virtual scan trigger. HTs are difficult to detect because of the rare activation probability. This trigger can greatly increase the probability that the HT is triggered by a higher transition probability. The simulation results show that the HTs detection is significantly improved and HT activation time is reduced.

Zhou et al. [103] proposed a low-cost and efficient technology that utilizes logic gates. Based on the fact that the transition probability of a logic gate is self-influenced, this method increases the net transition probability and accelerates HT detection by the 2-to-1 MUX as a test point. Experiments have shown that this method helps to activate HT, which makes testers find HT easily.

Lecomte et al. [104] presented a way that adding a network sensor sensitive to supply voltage Vdd. Those sensors are placed so that it covers the whole IC surface. They regarded HTs detection problems as fingerprint recognition of the supply voltage (Vdd) over the whole surface of an IC problem. Comparing the fingerprint of normal ICs with it of the unknown ICs identifies the counterfeit and malicious ICs.

Zhang et al. [105] suggested a hardware trust verification technology—VeriTrust, for detecting the HTs of the design stage. HTs are usually activated by input in a dedicated trigger, so it is not sensitive to the test trigger signal. VeriTrust automatically identifies these potential HTs by the checking and verifying module. The effect is not ideal although these HTs can be detected. The HTs cannot be activated in most cases.

Xue et al. [106] introduced a new microelectronic HTs detection circuit based on timing analysis to detect combined and sequential circuits. Because the inserted HT will result in additional capacitance, the test path will generate more delays in charging and discharging. This method compares the output signal from the calibration CUT delay time (t) with twice the time (2t) to facilitate HT detection. To compensate for the influence of external factors on HT detection, a ring oscillator is proposed in order to estimate the environmental change.

Courbon et al. [107] proposed an efficient HT detection method with electron microscopy scanning imaging. This method uses the electron microscope to scan imagery for the single-layer structure in a chip and compares it with the standard design circuit, find out what has changed, which can accurately detect any modification, replacement, removal, or the addition of circuit door. Experiments showed that this method could accurately and efficiently identify HTs. Nevertheless, this method is at the expense of the circuit, and for the VLSI, the cost and required technical content will increase accordingly. As long as SEM imaging can complete the comparison of HT, it does not rely on complicated detection principles and processes, which is exactly where the convenience of the technology lies.

- **Runtime monitoring**

Runtime monitoring means that, while the IC is in operation, the monitor constantly monitors some specific objects in the circuit, thus indirectly detecting traces of HT activity. The advantage for this detection technology is that HT is found in the whole process of using IC, and it is not a one-time detection result, like other detection methods. It is quite difficult to activate all types of HTs, Runtime Monitoring method can classify attacks of HT and mark trust strengths based on the attack type [93,99]. Otherwise, Runtime Monitoring costs extra performance overhead to ensure chip security before chip deployment. Therefore, this article does not draw a specific category in the classification diagram of the HT detection method. Table 1 organized the following four references, including monitors and monitoring objects.

Mubashir et al. [108] designed an authentication scheme for the HTs by hijacking unauthenticated packages containing hardware Trojans on Network on Chips (NoCs). In the beginning, they inserted a tag in the packet. When receiving the packet, the destination DU recalculated the tag on the received packet and compared it with the original tag. If they are different, it considers a hijacked packet to be detected.

Bao et al. [109] proposed three innovative low-overhead methods for runtime HT detection. The first method uses the information from the thermal sensor output in the circuit and a hypothesis test to determine the presence of the HT. The second method utilizes the correlation among the sensors and uses a Kalman filter (KF) to maintain the thermal distribution of the circuit. The third method combines the leakage power information with the extended KF (EKF) in order to detect the thermal distribution of the circuit. The results indicate that the three methods proposed in this paper can detect Trojans efficiently and quickly.

Khalid et al. [110] introduced a general method to identify potential HTs, which uses the formal hardware verification to analyze HTs. They also designed a runtime monitor to model the HT formally. Although this method takes less area and power overhead, it increases by 5–30 times with the test time overhead.

Unlike the previous digital HTs, the analog trojan is very small and can only be triggered by rare events. Hou et al. [111] presented a detection framework. The principle is to protect a group of related signals. When an abnormal switching event occurs in these protected signals, a hardware interrupt request will be initiated. The method belongs to the runtime detection mechanism and it is practiced on the AMS circuit at present.

#### 5.2.2. Static Detection

Static detection is to perform feature extraction on circuit parameter information. These features can be selected and put into ML for training, and the classification results for normal and malicious circuits can be quickly obtained. The objects of classification can be circuit images, netlists, changing points in the circuit, physical parameters, etc. ML handles extremely large data sets, which enables static detection to cope with VLSI detection, while maintaining high efficiency. At present, ML classification based on netlist also realizes positioning.

Bao et al. [112] proposed a new method that combines ML with HTs detection. They convert the circuit model into an image and divide the image into smaller, easier-to-handle meshes. They utilize circuit image features to train the classifier and obtain decision boundaries. Based on the v-SVM decision boundary of each layer, the mesh of each layer on all chips can be classified as a malicious circuit or a normal circuit.

Xue et al. [113] set the HT detection problem into binary classification. Subsequently, they experimented on analog circuits using a variety of classification algorithms, such as Bayes Net, Logistic, SMO, LWL, etc. Their method can determine whether a circuit includes HTs in a short time.

Jain et al. [114] presented an HT detection method that combines reverse engineering (RE) and SVM algorithms. This means it can avoid damage during common RE inspections. They extract features from known standard chips through RE and distinguish unknown chips with the learned SVM classifier.

Dong et al. [55] established a framework for HT recognition at the gate level and the register level. An optimized decision tree algorithm in ML is used to extract features in the netlists of the circuit, and finally classifies the HTs of the above two levels. The results of the experiment are quite excellent in TPR and TNR. Additionally, this research team proposed a novel framework that is based on boundary network and also applied ML methods to detect HT [115]. The framework breaks through the limitation of image localization in the static analysis method and directly locates the HT through the netlist.

Elnaggar et al. [116] developed a runtime detection technology based on ML, which detects HT in the core of the microprocessor. Monitoring the data flow of performance counters in a non-intrusive way to detect abnormal behavior when the HT is activated. Anomaly detection based on change points can collect the tracking of performance counters, which can distinguish change points due to concept drift or trojan activation. It is to use the unsupervised clustering algorithm in ML in order to classify these change points.

Lu et al. [117] developed an unsupervised learning mechanism to get rid of the golden chip to find HT. Use the testing data to calculate the uncertainty of the binary information flow generated in IC, bring the calculated results into the clustering algorithm for analysis, and choose the logic gates with lower uncertainty as candidates for HT.

Shayan et al. proved that the valve HT can be realized by maliciously reducing the source of pressure on the biochip [118]. The ML detection method was applied to the FPVA biochip with a matrix structure [119]. The on/off state of the valve and whether the chamber was filled are expressed as binary vectors, and these vectors were put into the ML trainer as a dataset, and three types of valve-based attacks were distinguished in turn.

#### 5.2.3. IP Verification

IP verification is the method of detecting outsourcer’s IP core. There are currently three authentication schemes for trusted IP. The first approach determines the credibility of the IP core by test and identification. The second scheme is to obtain the trusted IP as much as possible. The third method relies on a new paradigm of expert-system-based IP analysis [120,121].

Chen et al. [122] extended the FASTrust framework for feature analysis of HTs in third-party IP cores and use standard cell libraries to transform third-party IP cores into the gate-level descriptions. A variety of feature matching algorithms are then invoked in order to detect the HT. The final test results in a candidate HT report can be manually screened by the operator.

Singh et al. [121] introduced an empirical third-party IP core analysis method. Based on the known IP core specification and its RTL code, they judge whether the difference between RTL and its original trusted description through information matching and infer the relationship betwee RTL and the specification document structure block by block.

Love et al. [123] proposed a novel framework for promoting trusted IP cores. This framework draws on the Carrying Proof Code (PCC), which allows IP consumers to verify security-related properties through direct calculations. IP vendors and consumers can determine these security properties without the exact features. Similarly, consumers can easily identify whether the IP core is consistent with the agreed attributes.

### 5.3. HTs Detection Techniques in Post-Silicon

#### 5.3.1. Optical Detection

The optical detection method is similar to Side-channel signal analysis. It judges whether the circuit contains HTs based on signals, such as light and heat that are generated by the IC during operation.

Song et al. [124] expressed that the energized active device will emit infrared light. This phenomenon led to the birth of a new technology that detects the light changes of the chip. This method relies on the Picosecond Imaging Circuit Analysis (PICA) tool, which is adapted to changes in the detected circuit. They finally proposed a case for screening HTs, which is about extracting the light changes in the emission map.

#### 5.3.2. Logic Testing

Logic testing is to generate multiple test data to meet certain rare activation conditions in order to stimulate HT. This approach involves applying the stimulus response to the ICs and comparing the test response to the expected response from the simulation calculations. If the responses of IC are affected during this test procedure, then HTs will be detected [86]. There is the main challenge in a logic testing based approach for VLSI, which makes the generation of an exhaustive set of test vectors to detect all possible HTs computationally infeasible [93].

Wang et al. [125] analyzed how to systemically construct rare combination conditions so that a malicious circuit with the desired triggering probability can be synthesized accordingly. They used the cube production method to design a compact and rare model. The mode can cover joint events combined series of single rare events and effectively activate the HTs.

Zheng et al. [126] proposed a kind of HT detection technique that is based on probability signatures. There is the logic test method, which judges whether HTs are inserted by comparing the probability signature of the tested circuit with the probability signature of the original circuit. The experiment indicates the method can detect all the HTs in the contaminated circuit, but they need a golden netlist sample as a reference.

Bazzazi et al. [127] introduced a method based on logic testing. They divided the main circuit into sub-circuits. In each sub-circuit, choosing some specific nodes. Obtaining the nodes with the greatest similarity by the output of these specific nodes. These similar nodes are candidate nodes whose relation corresponds to a value. The value is different between HT-free and HT-insertion. This method is scalable, overcoming the problems of noise and process variation.

Huang et al. [128] presented, in detail, a scalable statistical test generation method, which can generate a high-quality test set for creating highly relative activity in arbitrary HT instances. It generated efficient test vectors with side-channel signal analysis for HTs detection. They utilize the correlative switching of the HTs in the whole circuit to indicate the sensitivity of the side-channel signals. The statistical test patterns can maximize the relative sensitivity of HTs detection under the noise of any process.

#### 5.3.3. Side-Channel Signal Analysis

The side-channel signal analysis compares the standard ICs with testing ICs in all kinds of side-channel signals, such as voltage, temperature, path delay, etc. Judging whether the testing is inserted HTs or not according to the data gap, it is obvious that the essence of this comparison method bases on the phenomenon that some side-channel signal parameters could manifest the extra circuitry, although it does not detect faults in the circuit containing HTs. Nevertheless, not only noise interferes with the detection of HTs, but excessive process disturbances in nanotechnology also make detection difficult for this analysis method [91]. The following Table 2 simply compared the literature about representative side-channel signal analysis.

Zhong et al. [129] proposed an HT detection method that was based on differential temperature matrix analysis. This matrix is the temperature image of all pixels on the thermal image of the circuit over some time. In the experiment, the author also added several interference modules to the circuit to interfere with the detection of the HT. However, the results show that, as compared with the differential matrix of the standard chip, the circuit inserted into the HT has a significant differential temperature rise.

Amelian et al. [130] suggested a new kind of side-channel signal detection technique that doesn’t modify the circuit design and the hardware overhead. Moreover, the common side-channel signal detection techniques in VLSI have an alternative path to test. They obtained the shortest path by programming. HTs can be found more easily by comparing the delays of testing circuits with standard circuits.

Zarrinchian et al. [132] introduced a new side-channel detection method that was based on path delay. They combine the latch structure with the circuit, and this latch structure can show the delay caused by all possible HTs. This method can solve the impact of process changes on normal circuits in traditional methods. At the same time, this method can reduce the influence of noise to compensate for the poor performance of the side-channel signal analysis technique.

Tang et al. [131] presented an HT detection and positioning technology that was based on thermal images, which can detect HTs with less than 20 gates. The golden chip simulated by the simulation software is compared with the target IC on the redundant thermal image, and the activity factor is extracted according to the thermal change of the trojan in the redundant thermal map. This method is 10 to 100 gates more accurate than power-based and electromagnetic-based side channel detection methods.

#### 5.3.4. Reverse Engineering

**Figure 12:**
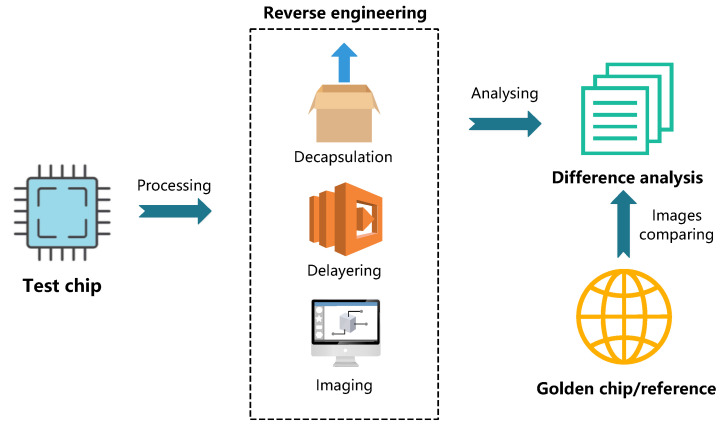
Reverse engineering extracts the chip structure images to detect HT.

Reverse engineering (RE) directly scan the physical structure inside the IC to be tested layer by layer, and then compare the extracted image after delayering with the golden chip/reference. This process can be visual inspection with the a microscope, or it can be used with auxiliary analysis (such as ML) in order to check HT. RE-based detection rates are higher, since HT is observed directly at the physical level. Destructively extracting IC information makes the method have certain limitations. Figure 12 shows the whole process.

Malik et al. [133] introduced two kinds of techniques for detecting HTs. The first one is functional analysis based on SAT and BDD reverse engineer ICs. Its purpose is that deriving a higher level of functionality of the IC by algorithmically analyzing IC’s netlist exposes the logic construction of the HT. The second method is that using a clustering algorithm performs statistical analysis on the chip simulation data in order to isolate the HT. The author combined the two techniques and acquired complementary advantages in benchmark circuit detection.

Bao et al. [134] used the previous several steps of RE to obtain internal pictures of IC. These internal pictures are the logic structure of the circuit and don’t represent any function of the circuit. Moreover, they use SVM to train images information and finally achieve the purpose of distinguishing the HT circuit structure and the normal circuit structure.

Bhasin et al. [135] presented an HT detection method that was based on direct visual inspection at the circuit layout level. Firstly, they compare the optical image of the test circuit with the optical image of the normal circuit. These images are extracted by destructive RE. Moreover, the authors measured the difficulty of HT insertion on the AES-encrypted IP core logic location and routing, and it turned out that HTs are difficult to play a role when the density reached 80%.

### 5.4. Classification of HTs Prevention Techniques

#### 5.4.1. Design for Trust (DfT)

There are two ideas for considering HT defense from a design perspective [87].

The first idea means hiding the function of the IC (obfuscation): the blur is mainly caused by the confusing design of the circuit so that the attacker can not find it. The structure of the original circuit, so the HT cannot be perfectly inserted into the original circuit, this method can also help to detect the HT while defending against HT attacks. Specific methods include:

- **Locking mechanism**

Inserting a built-in locking mechanism into the circuit to achieve the purpose of hiding functionality:

Roy et al. [136] suggested a technique, called EPIC (Ending Piracy of Integrated Circuits), to protect the chip by using a novel low-overhead combination chip locking system and a PKC-based chip activation protocol. Each chip must have a unique serial number when it is activated, so it is difficult to bypass the EPIC system without modifying the mask or IC.

Rajendran et al. [137] presented a joint circuit-architecture-level design approach. They used a variety of logic encryption techniques and circuit-level security techniques to construct a micro-architecture-level security module that prevents designers from inserting HTs and still maintains detection capabilities.

- **Obfuscation and filling**

Circuit layout obfuscation is the addition of circuit layouts that have no real meaningful function to an existing circuit design. These additional layouts make it impossible for an adversary to implant an HT with any offensive effect, due to the fact that they prevent an amateur attacker from identifying the circuit components that are actually function.

Cocchi et al. [138] suggested a circuit protection process that was based on circuit camouflage. Based on several existing circuit camouflage techniques, the author proposes three different novel types of digital camouflage logic units and designs a camouflage inventive filling method for circuit blur. The masquerading logic unit and the fill method can be separately used to prevent attacks from HTs.

Bi et al. [139] studied the existing safety technology and designed three typical circuit structures: camouflage gates, multi-state gates, and power regulators. The test results prove the feasibility of silicon nanowire FETs and graphene Sym FET in IC security.

Another idea about the Hardware Trojan is the limitation of the living space (filling): the filling is related with adequately utilizing the blank cells in the circuit, inserting the designed detection circuit (BISA), or removing the blank space, in order to limit the insertion of the HT space.

Ba et al. [140] introduced a method, called DFhT. The purpose of this method is to build a circuit layout with maximizing density. It uses functional cells instead of filled cells, so these fills are testable. Furthermore, functional cells will bring routing constraints, this paper also proposed an enhancement algorithm based on predecessors’ ideas to minimize the constraints imposed by padding.

Kan et al. [141] presented a functional circuit adding to the chip layout called obfuscated self-built authentication (OBISA). This circuit is connected to the original circuit and achieves the purpose of confusion based on reducing the filling space and self-detection. The gating mechanism and the network selection method proposed in this paper render the extra overhead of padding negligible.

#### 5.4.2. Split Manufacturing for Design (SMfT)

Front End of Line (FEOL) is usually high cost and untrustworthy manufacturers generally take part in the process. Subsequently, ship wafers from the former will be processed by trustworthy vendors of Back End of Line (BEOL). All of the above constitute an important part of the split manufacturing. If untrustworthy manufacturers do not involve the process of BEOL, they hardly determine the location of inserted HTs in the circuit [99]. This method is costly, and so far split manufacturing is still in the two-dimensional (2D) to three-dimensional (3D) integration area [142].

Vaidyanathan et al. [143] proposed split manufacturing after the Metal1 layer, which would confuse the underlying intent by hiding all of the gate connection. This paper also designed a split-made test chip, and the test results prove that this method is effective.

Hill et al. [144] first showed the standard manufacturing processes and split manufacturing processes of complex ICs. Split manufacturing alone cannot guarantee the safety of chip production, the paper also uses the cellTK [145], because it can almost prevent an attacker from inferring the key information of BEOL based on FEOL information. The dual-foundary proposed in this paper uses the geometry of Foundary A as the layout and the BEOL of Foundary B as the final BEOL.

Yang et al. [146] presented a 2.5D IC security-aware physical design flow for two attacks named proximity attack and SAT attack in 2.5D split manufacturing. This paper introduced a security metric for each of the two attacks and confused the offense by adding layout and functionality. Under the evaluation of these two metrics, the process that is proposed in this paper can effectively combat the two attacks.

Valamehr et al. [147] suggested developing a system using 3D integration. Arbitrary designs are divided into computational planes and control planes. The calculation plane is produced by an untrusted foundry, and a reliable foundry manufactures the control plane. This article also introduces a secure control plane that makes it possible to implement various security functions in a low-cost and efficient manner.

### 5.5. HTs Protection Methods Comparative Analysis

#### 5.5.1. HTs Detection Techniques Comparision

In the classification of the HTs detection methods in this paper, the Pre-silicon includes: Facilitate detection, Prevent HT Insertion, Static Detection, and IP Verification; the Post-silicon includes: Destructive, Logic testing, and Side-Channel signal analysis; the relative comparison is shown in Table 3.

The subsection compares these techniques from three aspects: Relative Tools, Characteristic advantages, Existed problems. There are detailed description in Table 3.

Facilitate detection and prevent HTs insertion belong to the invasive type. Tester needs to insert some auxiliary supplies into the circuits, increasing the cost is the biggest drawback, even cheap padding. Not only the present, but also the future, production cores use ICs on a large scale; nobody can afford such a high cost. However, the method is handy for protecting small-scale ICs without those attacks of modifying original circuits.

Static Detection is almost carried out from the design netlist of ICs. There are five abstractions of ICs, including System-level, RTL-level, gate-level, abstraction-level, and physical level. RTL-level and gate-level are the main directions of the test. Generally, static detection converts the HTs detection problem to binary classification problems in ML. Its advantage lies in the use of ML algorithms, it is not affected by the interference of external factors, researchers can experimentalize at any time and anywhere. These algorithms can mine information that cannot be manually obtained in the circuits. The method can handle almost any type of circuit just according to the netlist of the circuit. It is a relatively ideal way for VLSI HT detection at present.

IP verification is mainly aimed at the third party IP core in the chip production process. At the present stage, the main idea of detecting HTs in IP core is that it makes IP core fully run, find out suspicious signal (not frequently turned over), and determine whether there are HTs or not. However, it is not effective enough to detect large-scale IP core.

Optical detection is similar to side-channel analysis, except that it detects HTs based on optical images. This method can be combined with a variety of methods, not only from the circuit structure, but also from the perspective of the image. It is an extremely adaptable method. However, due to factors, such as image resolution, HT detection will be affected to some extent.

Logic testing is aimed at the rare activation conditions of the HTs, which are not affected by the external environment. The problem to be overcome by this test method is that HT could hide in vast circuit space, which manifests in being inadvisable for consuming countless computing power to find all possible HTs [91]. There is also the difficulty and key point of the logic test.

Side-channel analysis is the most popular technology to detect HT. There are a lot of testing signals, they can be used to determine whether a chip containing HTs easily. However, the influence of external factors, such as noise, this approach is powerless to subtle HTs in large-scale circuits. Additionally, the standard chip is a necessity. The cost is too high to get a standard chip. Additionally, circuit signal tests need all kinds of high precision instruments, which dramatically limits the experiment, the method is not the best choice now.

RE belongs to destructive testing, starting from the function description of the circuit to analyzing the circuit step-by-step. It will be able to obtain an HT-free chip, but the repeatedly detected chips hardly use. It is currently the most efficient way to obtain an available standard chip. However, each test takes a lot of time and workforce, the harvest is out of step with cost.

#### 5.5.2. HTs Prevention Techniques Comparision

The subsection compares the prevention techniques from two aspects: characteristic advantages and existing problems. There is a detailed description in Table 4. The HT prevention techniques can be divided into the DfT and SMfT.

DfT is generally by inserting additional circuit components or obfuscation functions or increasing the Layout Filling. However, since both technologies require additional circuitry, additional overhead is unavoidable. The biggest limitation of DfT’s approach is the high extra overhead. However, for some high-precision, low-volume production chips, the method is a wonderful defense option. Without the limitation of high overhead, this method can greatly improve the security of ICs.

The main idea of SMfT is that it divides the manufacturing of the chip into two parts. This method can effectively create physical isolation for the insertion of HTs. However, 2D manufacturing, 2.5D manufacturing, to 3D manufacturing still have drawbacks in process manufacturing. At the same time, SMfT has a long manufacturing cycle and greater manufacturing cost than DfT, and cannot be applied in the market on a large scale.

## 6. The Prediction of Future Trend

VLSI has become the main force in the global electronic device market. While HTs will be more precise, smaller, and more concealed, the detection techniques of HTs are is changing with each passing day.

**Figure 13:**
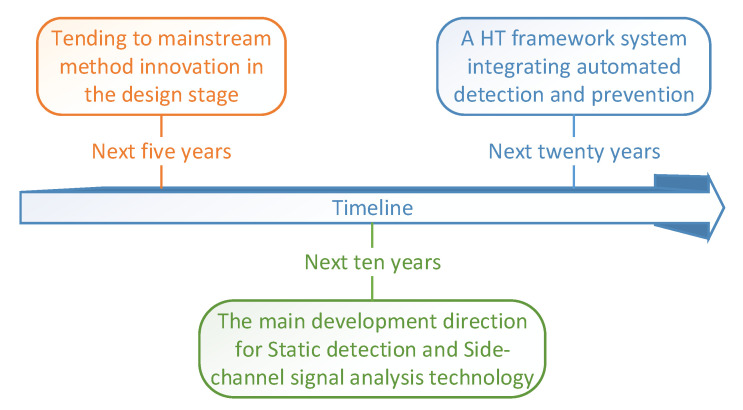
The future trend of HT.

By summarizing a large amount of literature, this paper predicts the future research trend of HT in Figure 13 and divided the overall trend of dealing with HTs into three phases for the next 20 years:

(1) There will be various new detection techniques based on existing techniques in the next five years. The relative researchers will go on innovating in the existing mainstream methods. In particular, the HT detection technology is more inclined to the design stage, the possibility of HT implantation at the fabrication stage is low.

(2) In the next ten years, the relevant studies will tend to static detection and side-channel analysis detection techniques. They are both efficient and accurate methods of detecting HTs. They can handle most parts of HTs in the marketplace. However, they are limited to the testing in individuals or small laboratories. Other stages of the IC cycle will discover new threat models and develop corresponding detection and prevention technologies.

(3) In the next twenty years a more complete system/framework will appear [55,122,148,149,150,151,152]. This ideal system/framework is an integrated automation HTs processing tool. It combines existing mainstream HTs detection methods with prevention methods to reduce the loss of a single technology defect. This tool not only saves the technical cost of processing HTs, but also greatly enhances the processing efficiency. It will inevitably be the mainstay of detecting HTs.

Based on a large amount of existing literatures, future research directions will mainly focus on HTs detection technology combined with artificial intelligence, golden-free techniques, prevention with detection, HTs in wireless networks, and specific HTs protection techniques. In the following content, the paper discusses the prospective development trends from six major research directions.

### 6.1. Development of HTs Design

At present, the design concept of mainstream HTs is developing to zero overhead, intelligence, and decentralization.

The semiconductor HT was designed by Becker et al. [153]. It is inserted by changing the doping polarity of existing transistors. This type of HT does not require additional circuit primitives, the appearance and functionality of the IC do not change when the HT is not activated. The HT is only activated when the temperature of the infected circuit reaches a certain peak. At that time, the polarity of the circuit is deflected, which results in an abnormality in the function of the IC. Presently, optical detection and side-channel detection techniques are almost impossible to detect that HT.

Yuan et al. [154] successfully steal the digital signals of the differential interconnects in the IC by inserting additional crosstalk sensitive loops into the circuit. The experiments show that, in the actual case of non-uniform media, the HT can effectively reconstruct the original state of the circuit. The trojan has a simple structure and no trigger module, and it can easily bypass the general detection means.

Zhang et al. [155] designed an optimized lightweight HT. The authors used Algebraic Failure Analysis (AFA) to determine the optimal failure model while streamlining the trojan’s capabilities flips only one bit of the circuit. In the test experiment, the trojan can easily obtain the DES key with only one glitch.

Hoque et al. [156] developed an HT that can tamper with data for static random access memory (SRAM). The trojan is inserted into the memory array and it has a unique triggering mechanism, which greatly increases the difficulty of detection. At the same time, the trojan will not show other bypass signal changes and will not generate other space overhead. Such an HT can almost avoid all mainstream trojan detection techniques.

Boraten et al. [34] designed an HT for Target Activation Order Payload (TASP). This trojan can perform deep data checks of the system covertly, it activates the ECC to schedule resources until the application crashes repeatedly.

Ye et al. [157] suggested the concept of the HT gray area, which is likely to be the direction of HT design in the future. The author uses ML algorithms and behavioral simulation techniques to detect trojans on Trust-Hub. Trojans that are undetectable by any of the two methods are gray areas. They also proposed the concept of a gray area boundary, but they are temporarily unable to determine the boundary.

Wang et al. [158] developed two advanced counting HTs, two types of HTs are based on Gray code and One-hot code, respectively. These two trojans are characterized by extremely simple structures, long-term, controllable incubation periods, and almost imperceptible consumption.

### 6.2. HTs Detection Techniques at Gate-Level

The static detection technique gradually the becomes popular because of its convenience and predictability. It does not require extra equipment and expensive costs. The combination of hardware security issues and ML algorithms makes the original complex detection problem solvable without the golden chip. As long as the features of the HT circuits are obtained, the required classifier can be trained for classification. However, the disadvantage of the method is difficult to build a perfect model for the ICs. For the features of the circuit, it is difficult to intuitively reveal the difference between the ordinary circuit and the HT circuit. The problem of HTs detection can only be handled by the experience and numerous samples in the black-box model of the ML algorithm.

Nevertheless, a set of representative circuit features can maximize the classification effect and perfectly dispose of HTs. Hence, the approach will inevitably dominate the main market for HTs detection in the future. Based on the proposed researches, many articles about the gate-level netlists have emerged in large numbers. The gate-level of the circuit is a significant stage before the circuit package. It completely describes the logical structure of the circuit but it does not consider the original function of the circuit. Therefore, in the future, one of the researches for the HTs will start from the gate-level netlist. The literature about static detection technique was published in the past two or three years.

The main purpose of Shen et al. [159] is that generating a valid test pattern detects hardware triggers that are triggered in the internal RTL code. They analyze the Verilog HDL code generation control flow graphs (CFGs) in the gate-level netlist and use the symbolic execution and metamorphosis tests to generate the expected test vectors. They used the data on Trust-Hub as a test set and proved the effectiveness of the method.

Sebt et al. [160] presented an effective net magnetic susceptibility index, which can perform low-cost calculation and analysis on the controllability and observability parameters of various networks in the circuit, obtained a netlist vulnerability map, which can manually select suspicious circuits. Finally, the HT network is selected twice with the classification algorithm based on the idea of region division. Experimental results show that the method can handle large-scale circuits, which will provide ideas for HT detection in ultra-large-scale IC.

Nourian et al. [161] suggested an HT detection technology that combines genetic algorithms and logic testing techniques. The author generated an initial test vector that is evaluated with a fitness function that consists of a netlist, a parameter of the genetic algorithm, and a threshold. It calculates the conversion probabilities of all nodes and uses the threshold to identify rare nodes. Calculating the SCOAP parameters. Finally, using the propagation of the genetic algorithm generates test vectors. The paper made a contribution to propose a suitable fitness function.

Chen et al. [162] introduced a one-shot HTs recognition technology based on gate-level circuit structure characteristics. Firstly, extracting the HT structure template from the netlist, making a critical statistic according to the matching degree of submodule and template in the test circuit. They put forward to the scoring method and the abnormal score method in order to verify whether there are HTs in the circuit or not. The method has the advantages of low false alarm rate and low overhead and expandability, which supplements the existing library of the HTs feature. However, the method does not recognize HTs with special structures and it is powerless to HTs with low probability activation.

Salmani et al. [163] developed a novel HT detection and a recovery detection method by gate-level netlists without reference. The approach makes use of the distance between special clusters, which is between the HT circuit and normal circuit in controllability and observability, to conduct unsupervised cluster analysis and feature classification. The experiments showed that the method did not require the activation of HTs and not the golden netlist. It can achieve a zero error rate and zero false alarm rate detection. However, the method cannot accurately detect the actual number of HTs, and can only judge whether there are HTs to a certain extent. Therefore, the method of HT identification should be improved.

Xie et al. [164] proposed an HT identification technology by studying the properties of gate-level netlists. The approach extracts the controllability and observability of the netlist by the k-means and the support vector machine (SVM). The method has a high degree of detection and robustness and does not require any test mode application. However, there is still room for improvement in the selection of feature parameters and the construction of classification algorithms. The k-means clustering method is sensitive to the initially selected cluster center point, different random seed points cause completely different cluster results. The classification result of HTs is different.

### 6.3. HTs Detection with Golden-Free Techniques

Destructive testing is gradually outdated, due to time-consuming overhead and expensive costs, but it is an undoubted way to obtain a golden chip. For some small-scale chips, destructive testing still has considerable demand.

Even though noise can interfere with the side channel detection, the techniques rely on the golden chip, and their efficiency is well-known. In recent years, most scholars have continuously devoted to improving the main drawbacks of these methods, even without the golden chips in some methods. If the shortcomings of the side channel detection techniques are overcome, the method will be extremely efficient in regards to HTs detection problems.

Xue et al. [8] proposed a method to generate antagonistic data in opposition to the modification of third-party circuit data. Moreover, an HT detection method that is based on the clustering sensor is proposed. The author used an unsupervised learning method to select features, so that it reduces the impact of operation status and process noise on HT detection. The method eliminates the need for standard chips. Experiments on the ISCAS89 standard chip show that the method can resist the HT attack and keep high-precision for the HT detection.

Xue et al. [165] introduced a self-referencing HTs detection based on the overall leakage of the circuit caused by the insertion of the HT. The author divides the circuit into segments and calculates the operating variation scaling factor of all segments in order to determine whether a circuit is HTs-inserted or not.

Zhang et al. [166] suggested two power signal description methods for data-activated processor trojans: The first method combines the contradiction equation and the differential bit power to calculate the power of each bit. The second method uses a detection algorithm to statistically analyze the error bounds. Both methods have almost no detection error and can successfully detect HTs.

Oya et al. [167] suggested a classification method that was based on the score. The method extracts the characteristics of HT networks, marks, and makes a statistic with corresponding scores, and then uses a scoring threshold for classification. The advantage is that it can identify malicious circuits without a golden netlist. However, the accuracy of the detection depends on the availability of the HT features, so that the accuracy of the test results is low.

He et al. [168] combined electromagnetic spectrum modeling and statistical analysis of side-channel signals to make an HT detection approach. They collected data at the RTL level and use the simulated electromagnetic spectrum as a standard reference model, focusing on frequency domain signals.

### 6.4. HTs in Wireless Network

In fact, after more than a decade of research, the defense and protection technology of digital ICs has been quite mature and effective [5]. With the development of wireless communication technology, electronic devices are increasingly relying on wireless, which gives HTs more opportunities to attack and brings more new challenges. The HTs defense in the wireless network will be a significant task in the future.

Jin et al. [63] studied HTs in wireless encryption circuits. The HT adds a structure to the signal to be transmitted to issue commands or leak information. The authors consider the ignorance of these structures, using advanced statistical analysis of various circuit parameters (amplitude, frequency, phase, etc.), they also combined with principal component analysis in order to effectively detect trojans in the test circuit. An effective attack cannot be performed because an attacker cannot know what the designer will analyze.

Liu et al. [36] simulated two types of HTs that exist in power amplifiers and RF pulse generators. These HTs will reveal keys in the wireless network. Both HTs have PMOS transistors on the output/input. When the key bit is 0, the PMOS transistor is turned on; when the key bit is 1, the PMOS transistor is turned off. The effects of the HTs on the original circuit are minimal and they will not affect the operation of normal circuit.

### 6.5. Specific HTs Protection Techniques

In addition to the current mainstream HTs detection and defense methods described above, some methods are difficult to categorize, because they are designed for specific HTs. With the development of information technology, there will be more specific HTs, and preventing these HTs will be another difficult problem in the future. In addition to some special HT protection methods, there are also HT protection technologies for emerging chips.

Wang et al. [27] proposed a new HT that is more difficult to defend. At the same time, they proposed a feature matching method that is based on information flow (IFT) tracking, which can obtain the information of HT at RTL.

Rajamanikkam et al. [169] focused on an important threat in 3PIP: the architecture state preservation HT (TASP), and proposed three complementary techniques to detect the HT at a lower cost.

Some are extremely novel methods: Liu et al. [170] analyzed the combined structural characteristics of the HT and the main circuit and proposed a new HT detection technology. A feature database is constructed by extracting the structural characteristics of the combination logic and sequential logic in the group HT, and the author used the feature matching method to extract all of the features from the circuit structure.

Babu et al. [171] combined the side-channel detection method and the static detection method to calculate the power variation of the ISCAS85 and ISCAS89 reference circuits. They used the power profile training machine of the HT circuit and the normal circuit in order to realize the effective classification of the HT circuit.

Rooney et al. [172] successfully detected the dormant and activated HTs based on existing device technologies, such as power analysis reports, side-channel analysis, and so on. The author set a clear boundary for the detection of different HTs to reduce the loss of detection technology that cannot handle certain HTs. They will study more testing methods in the future.

Fyrbiak et al. [149] demonstrated a static analysis technology based on boolean function analysis and graph neighborhood analysis. The implementation of this technology is done by a plug-in of HAL and it does not depend on golden chips. They also used the HAL framework to implant HTs into the gate-level netlist and realized the universality of HAL.

Alsaiari et al. [173] used the reconstruction assertion checker (RAC) on SoC to detect HT, which is achieved by integrating the magnetron into the SoC, but the circuit needs to be redesigned. RAC can realize the possibility of updating the HT database on the SoC, because the previous assertion checker lacks reconfigurability and cannot deal with the new HT flexibly. The authors proved the effectiveness for DoS and information leakage HT, area cost, and power cost were 3% and 1%.

He et al. [53] carried out the work on the memristor-based HT for the AI chip, and developed the HTcatcher technology similar to side-channel analysis, which reduces the memory overhead in feature calculation by at least one-quarter.

Shayan et al. [118] designed a valve-based HT attack model on FBMB, and proposed that the ultimate pressure test on the valve should be carried out in order to deal with the HT.

Currently, there is no official definition of the above approaches. The paper shows that these methods are expected to be studied and exchanged by other scholars.

### 6.6. Benefited Application for HTs

Generally, HTs were derived from the negative aspects. They were parasitic on the credible and legitimate circuits, so that thy showed various malicious ways to the circuits. However, the HT is not completely harmful to the hardware. The beneficial applications for HTs develop beneficial effects on hardware security. HT-like watermarking [174] is a technology based on the features of the HT.

HT-like watermarking can be used to ensure the reliability of IP. However, traditional IP has poor concealment and it is easily wiped by attackers. Mohammed et al. proposed the new-style watermarking technology. According to the low overhead and the difficult detection for HTs, the watermark is designed as a small circuit similar to HT. Hence, it is difficult for an attacker to detect the existence of the watermark, the watermark is only found by two specific triggers.

Although this novel idea is relatively rare at present, it is enough to show that the HT can be used flexibly in other directions. HT is not blindly regarded as a bad thing. New applications of HTs will become increased in the future.

## 7. Conclusions

This article summarizes and discusses numerous HT detection and prevention methods, and it proposes an HT threat framework based on the degree of threat and destructiveness, and attaches importance to HT security on four layers. Additionally, the discussion about HT on emerging chips is a feature of the article. It breaks through the previous review that only focuses on digital/analog circuits while ignoring the safety generalizations of other chips (Biochip, AI chip, and RF IC). These chips have broad market prospects in the future, and it makes sense to initiate hardware security research on the corresponding platforms.

Section 6 predicts the trend for future HT from design to detection and prevention methods. With the development of the overall IC environment and expansion of demand, there are still open issues and challenges on ICs. These points are, as follows: (1) at present, the gate-level detection mainly relies on the ML detection method, which has high requirements on the feature extraction of the circuit. Selecting effective parameters among many features is the key to affecting the detection rate. How to find accurate and suitable features is a difficult subject for ML to use on the gate-level netlist, which also needs to be further explored by later researchers. (2) In recent years, the golden-free detection has had some detection schemes that are separated from the dependence on the golden chip, but the adopted methods still retain the golden concept; in other words, it is to compare the golden reference. Not only is there no inherent thinking of breaking through the comparison of reference objects, but some detection methods are not mature enough for commercial use. (3) The rapid development of IoTs environment and the urgent need for smart devices to rely on wireless sensing technology, coupled with the vigorous laying of infrastructure in the 5G field in recent years. The huge wireless application scenario should be adapted to the HT security framework under the same network scale. However, the current research is still in its infancy and it is more limited to small wireless networks, such as WiFi. (4) The special HT protection technology stays within the scope of the ICs. When compared with the traditional IC circuit, the emerging chips (AI chips and biochips) have special physical structures, which will also bring the unique security technologies of the corresponding platform. Nowadays, there are very few HT achievements in this field, and there are still many blanks to wait for breakthroughs. (5) There are still very few benefiting applications for HT in research at present, and there is still a lot of potential space for mining in this area for future research. It aims to reveal the advantages of HT, so that it can serve hardware security, not just threats, and destruction.

The above open issues are not only a challenge, but more of an opportunity. If a complete detection and prevention system/framework can be implemented in the future, HTs will be no longer scary. It is hoped that the paper provides basis and guidance for future research.

## Figures and Tables

**Table 1:** Monitoring properties of four references.

Monitoring Object	Monitor	Reference
Tag for packet	Routing computation module	[108]
Temperature in IC	Extended Kalman filter	[109]
Risky circuit path	Binary counter, 2 to 1 MUX, 8 to 1 MUX, controller	[110]
A set of concerned signals	Toggle event counter	[111]

**Table 2:** Comparison for physical parameters in Side-channel signal analysis.

Reference	Physical Parameters	Advantage	Disadvantage
[131]	Based on thermal	1. Achieving the detection and positioning of HT 2. Superior to the power and electromagnetic detection method	Rely on reverse engineering tools to extract a golden reference
[130]	Based on path delay	1. No changes to the circuit structure 2. No hardware overhead 3. Only detect the shortest path	Incomplete interference handling in the process variation
[132]	Based on path delay	1. Self-reference detection 2. Not subject to attack type 3. The impact of process variation is minimal 4. Any path in the circuit can be used for measurement	1. Excessive area overhead 2. Preprocessing relies on calculated iteratively
[124]	Based emission of light	No damage to chip structure	1. Limited spatial resolution 2. Rely on excellent equipment imaging accuracy

**Table 3:** HTs detection techniques comparision.

Detection Stage	Detection Technology	Relative Tools	Characteristic Advantages	Existed Problems
Pre-Silicon	Facilitate detection	Special circuit components, such as I/O ports and ring oscillators	Enhancing the detection efficiency of side channel detection or logic detection	Additional equipment overhead or circuit area overhead is required, sometimes the circuit design needs to be changed
	Static detection	Chip netlist	High detection accuracy, flexible in spection operation, no require of standard reference, suitable for VLSI	It is hard to extract the HT features used for detection
	IP Verification	IP cores	Prevent the third-party vendors attacking	Limited scale of the detection
Post-Silicon	Optical detection	Picosecond Imaging Circuit Analysis (PICA) tool, Optical instrument	No need to carry out complicated electrical test and logic test, and the test result is more intuitive	Highly time consuming for large scale circuits. High resolution for small circuits
	Logic testing	Automatic test platform	The detection method has high stability and is less affected by noise. It is suitable for HT chips of specific pin-triggered	Large number of measurement vectors, long testing time, limited application range
	Side-channel signal analysis	Precision oscilloscope. High precision temperature detector. High-precision power analyzer. High-precision spectrum analyzer and so on	The detection accuracy is high and the limited conditions are less. The HTs are not required to be triggered. As long as the circuit works, the signal can be collected. Be suitable for small scale in tegrated chips	Needing of highprecision equipment, effect of measurement accuracy, needing of standard chip reference. Effect of the external factors
	Reverse Engineering	Electronic scanning microscope, Optical scanning microscope, Voltage contrast imager, Circuit Analyzer	High detection accuracy, suitable for simple structure data	The detection time is long, the cost is huge; The chip is damaged, the requirement to the measuring equipment is higher

**Table 4:** HTs prevention techniques comparison.

Detection Stage	Detection Technology	Characteristic Advantages	Existed Problems
DfT	Layout Filling	HT in self-detection circuit, reducing HT insertion space	Not applicable to integrated cir cuits with complex structure functions, which brings addi tional unnecessary overhead and affects chip performance. Unable to block parametric HT
	Static detection	Chip netlist	High detection accuracy, flexible in spection operation, no require of standard reference, suitable for VLSI
SMfT	Optical detection	Picosecond Imaging Circuit Analysis (PICA) tool, Optical instrument	No need to carry out complicated electrical test and logic test, and the test result is more intuitive
	Logic testing	Automatic test platform	The detection method has high stability and is less affected by noise. It is suitable for Hardware Trojan chips of specific pin-triggered
